# Virus-Mediated Cell-Cell Fusion

**DOI:** 10.3390/ijms21249644

**Published:** 2020-12-17

**Authors:** Héloïse Leroy, Mingyu Han, Marie Woottum, Lucie Bracq, Jérôme Bouchet, Maorong Xie, Serge Benichou

**Affiliations:** 1 Institut Cochin, Inserm U1016, 75014 Paris, France; heloise.leroy@inserm.fr (H.L.); mingyu.han@inserm.fr (M.H.); marie.woottum@inserm.fr (M.W.); 2 Centre National de la Recherche Scientifique CNRS, UMR8104, 75014 Paris, France; 3 Faculty of Health, University of Paris, 75014 Paris, France; 4 Global Health Institute, Ecole Polytechnique Fédérale de Lausanne (EPFL), 1015 Lausanne, Switzerland; lucie.bracq@epfl.ch; 5 Laboratory Orofacial Pathologies, Imaging and Biotherapies UR2496, University of Paris, 92120 Montrouge, France; jerome.bouchet1@parisdescartes.fr; 6 Division of Infection and Immunity, University College London, London WC1E 6BT, UK; maorong.xie@ucl.ac.uk

**Keywords:** virus, cell-cell fusion, syncytia, virus spreading

## Abstract

Cell-cell fusion between eukaryotic cells is a general process involved in many physiological and pathological conditions, including infections by bacteria, parasites, and viruses. As obligate intracellular pathogens, viruses use intracellular machineries and pathways for efficient replication in their host target cells. Interestingly, certain viruses, and, more especially, enveloped viruses belonging to different viral families and including human pathogens, can mediate cell-cell fusion between infected cells and neighboring non-infected cells. Depending of the cellular environment and tissue organization, this virus-mediated cell-cell fusion leads to the merge of membrane and cytoplasm contents and formation of multinucleated cells, also called syncytia, that can express high amount of viral antigens in tissues and organs of infected hosts. This ability of some viruses to trigger cell-cell fusion between infected cells as virus-donor cells and surrounding non-infected target cells is mainly related to virus-encoded fusion proteins, known as viral fusogens displaying high fusogenic properties, and expressed at the cell surface of the virus-donor cells. Virus-induced cell-cell fusion is then mediated by interactions of these viral fusion proteins with surface molecules or receptors involved in virus entry and expressed on neighboring non-infected cells. Thus, the goal of this review is to give an overview of the different animal virus families, with a more special focus on human pathogens, that can trigger cell-cell fusion.

## 1. Introduction

Viruses are acellular organisms in which the genomes consist of RNA or DNA nucleic acid and which obligatory replicate inside host cells. Using cellular machineries, viral components are synthesized and then assemble into particles called virions, which serve to protect the genome for subsequent transfer to other target cells for virus dissemination. In addition, numerous viruses have a lipid bilayer external envelope that surrounds the viral particle. The process generally described for animal virus dissemination between permissive cells is related to release of cell-free viral particles that diffuse from infected cells and subsequently attach and enter new target cells for virus replication. The first step of this process is the recognition of cellular attachment molecules and receptors expressed on the surface of the target cell by virus-encoded proteins found on the surface of the virus particle. Recognition and binding to cellular receptors is followed by virus entry for replication. However, many viruses, including some viruses that are pathogenic for humans, can also move between cells without diffusing through the extracellular environment by cell-to-cell spreading. Virus cell-to-cell spread may not only facilitate rapid viral dissemination but may also promote immune evasion and contribute to pathogenesis.

Several distinct mechanisms for virus cell-to-cell transfer and spreading have been described, but some viruses can use the fusogenic capacity of some viral proteins, usually involved in virus entry, expressed at the surface of infected cells to trigger cell-cell fusion between infected virus-donor cells and neighboring target cells to form enlarged multinucleated cells, often called syncytia. Three main different classes of viral fusion proteins have been structurally described depending the conformational structure and mechanism use for fusion of the viral bilayer envelope with the cell membranes [1]: class I, with a characteristic α-helix trimer (as in HIV-1 transmembrane gp41); class II, with a β-sheet-based elongated ectodomain (as in dengue virus glycoprotein); and class III, composed of an α-helix and β-sheet combined ectodomain (as in rabies virus G glycoprotein). In addition, a fourth class of fusion proteins corresponding to the FAST protein family of some non-enveloped Reoviruses has been also described. Thereby, virus-induced cell-cell fusion and syncytium formation are mainly mediated by specific interactions of certain viral fusion proteins with surface molecules or receptors expressed on neighboring non-infected cells.

Several viral families, including some human pathogens, have evolved the ability to trigger cell-cell fusion to form syncytia between individual infected cells and neighboring uninfected or infected cells. Even if the specific role of these virus-induced syncytia for pathogenesis during the natural course of some viral infections is still discussed, these infected multinucleated cells can show high capacity of viral production and improved capacities of motility or survival, at least when recapitulated in vitro in tissue-culture assays. With the notable exception of some non-enveloped viruses of the Orthoreovirus and Aquareovirus subfamily of *Reoviridae* that use this cell-cell membrane fusion process and syncytium formation for virus dissemination, all other animal viruses able to use cell-cell fusion belong to families of enveloped viruses. For example, HIV-1 and SARS-CoV-2, the two major viral pathogens responsible for the global pandemics of AIDS and COVID-19, respectively, can induce cell-cell fusion and syncytium formation as largely evidenced in tissues, such as brain and lungs, of infected patients. Similarly, the presence of infected multinucleated giant cells in skin lesions has long been recognized as the hallmark of infection by some *Herpesviridae*.

Here, we will review the data of the literature regarding the main families of animal viruses able to trigger cell-cell fusion, and discuss about the mechanisms of this virus-induced cell-cell fusion process, and how this process could contribute in intercellular virus spread, virulence, and virus persistence.

### 1.1. Herpesviridae

*Herpesviridae* are a large and diverse family of enveloped double-strand DNA viruses which have a very broad host range and can establish long-life persistent infections. The human *Herpesviridae* family is divided into three subfamilies: α-herpesviruses, composed of herpes simplex virus types 1 and 2 (HSV-1 and HSV-2, also called HHV-1 and HHV-2, respectively), and varicella-zoster virus (VZV, or HHV-3), are fast-growing cytolytic viruses that establish latent infections in neurons; β-herpesviruses, including human cytomegalovirus (HCMV, or HHV-5) and human herpesviruses 6 and 7 (HHV-6 and HHV-7), are slow-growing viruses that become latent in secretory glands and kidneys; and γ-herpesviruses, composed of Epstein-Barr virus (EBV, or HHV-4) and human herpesvirus 8 (HHV-8, or Kaposi sarcoma-associated herpesvirus) are latent in lymphoid tissues, with a high restricted host range.

The formation of multinucleated giant cells (MGCs) (or syncytia) following Herpesvirus infection in their natural hosts has been well documented for a long time [2,3,4]. The presence of MGCs in skin lesions has indeed long been recognized as the hallmark of Herpesvirus infection [5] and could be used as diagnostic for Herpes simplex keratitis in eyes [6]. Similarly, MGC formation is also a cytopathologic feature of Herpesvirus infection in the lower respiratory tract [7]. The extent of Herpesvirus-mediated cell-cell fusion leading to MGC formation is related to the identity of the Herpesvirus but also to the infected tissue: VZV infection results in extensive syncytium formation in skin lesions [8], while HSV-2 induces limited syncytia consisting of only a minor population of infected cells in the skin lesions [9]. However, the significance of Herpesvirus-mediated cell-cell fusion for virus replication and spreading in vivo remains unclear. In in vitro tissue culture, the degree of cell-cell fusion mediated by different clinical isolates and laboratory-adapted strains can significantly varies [10,11]. For example, HSV-1 primary isolates cause limited cell-cell fusion [12], whereas viral variants from laboratory stocks induce extensive syncytial formation in tissue culture [13,14].

Herpesviruses enter host cells by enabling membrane fusion of viral envelopes with host cellular membranes, which either occurs at the plasma membrane or in endosomal compartments. This viral entry process is cell-type dependent and depends on the identity of the Herpesvirus. The viral core membrane fusion machinery required for cell-free virus entry but also for cell-cell fusion induced by herpesviruses consists of the viral glycoprotein gB, a type III viral membrane fusion protein that forms homotrimers, and the heterodimer gH/gL, which are conserved envelope proteins among all Herpesviruses [15,16]. gB is a major determinant of Herpesvirus infectivity both in vitro and in vivo [17,18], while the gH/gL heterodimer can interact with the gB and is required for its fusogenic function [19]. The requirement of the gB homotrimers and gH/gL heterodimer for virus entry into target cells is a highly conserved function among all Herpesviruses [20]. The general process for virus cell-fusion of Herpesviruses first involves activation of the gH/gL heterodimer upon binding to the cellular receptors leading to activation and conformational change of gB [20,21] for insertion of its fusion loops into the host cell membrane, followed by the refolding of gB to drive merge of the viral envelope with the cell membrane [16]. The core fusion machinery is required both for entry of cell-free Herpesviruses into target cells and Herpesvirus-induced cell-cell fusion [15,16,17,18,19,20,21,22]. However, the mechanism of Herpesvirus-induced cell-cell fusion is still poorly understood and is highly cell type-dependent, suggesting that specific cellular cofactors may play important roles in this process. For example, VZV induces extensive syncytial formation of primary keratinocytes but poorly causes cell-cell fusion of primary fibroblasts [8].

In addition, the membrane fusion process sometimes also involved non-conserved membrane glycoproteins specific to each Herpesvirus, which can bind to host cell-specific receptors. For example, HSV-1 and HSV-2 express a non-conserved glycoprotein gD that binds to different host cell receptors depending of the target cell [23]: nectin-1 and nectin-2, cell adhesion molecules expressed on neurons and epithelial cells [24]; the Herpesvirus entry mediator (HVEM), also called Herpesvirus entry mediator A (HveA) or tumor necrosis factor receptor superfamily member 14 (TNFRSF14), expressed on activated lymphocytes [25]; and a non-protein receptor called soluble 3-O-sulfated heparan sulfate [26]. HCMV expresses a glycoprotein gO, as well as 3 small glycoproteins encoded by viral *UL128*, *UL130*, and *UL131* genes, to mediate receptor binding and regulate cellular tropism, but the respective host cell receptors for these additional glycoproteins remain unclear [27,28]. HHV-6A and HHV-6B also express a glycoprotein gO and additional gQ1/gQ2 proteins to engage the CD46 cell surface receptor on human target cells [29,30], but some observations indicated that HHV-6 type B can also use human CD134 as an alternative receptor for viral entry [31]. EBV expresses a soluble gp42 to bind MHC class II receptor HLA-DR1 on B lymphocytes but uses gH/gL heterodimer to directly bind cell surface integrins, such as αvβ5, αvβ6, or αvβ8, for viral membrane fusion [32,33].

We will now focus on HSV and HCMV for which the process of cell-cell fusion has been best characterized. For HSV (HHV-1 and HHV-2), virus-induced cell-cell fusion and MGC formation for transfer of virus particles from infected cells to uninfected neighboring cells has been initially proposed, in addition to cell-free virus infection, as another mechanism by which viruses spread between host target cells [34,35]. HHV-1 utilizes cell-to-cell spread to move directly from mucosal epithelial cells, the initial site of infection, into nearby sensory neurons, where the virus establishes a latent infection. MGCs (also referred as poly-karyocytes) were first described in skin lesions of HSV-infected patients many years ago, and have even been used for diagnostic purposes of HSV infection in skin biopsies [5]. Similarly, early studies described the formation of MGCs when human epithelial cells were infected with primary HSV strains isolated from eyes or the oral cavity of infected patients [36]. These poly-karyocytes formed both in skin lesions and in tissue culture, containing no more than ten nuclei, have been referred to as the ‘small MGCs’ [37]. However, many HSV variants that cause much more extensive cell-cell fusion in tissue culture were readily isolated from laboratory viral stocks [2,3,4,5,6,7,8,9,10,11,12,13]. These resulting MGCs may contain hundreds or thousands of nuclei, and result from mutations in one or more viral genes [22,23,24,25,26,27,28,29,30,31,32,33,34,35,36,37,38]. For example, truncations or single-point mutations in the cytoplasmic tail of the gB glycoprotein can cause extensive syncytia formation in tissue culture [39,40,41]. Cell-cell fusion by syncytial variants requires the activity of the specific glycoprotein gD in addition to the core fusion machinery including gB and the heterodimer gH/gL. The gB and gH/L core fusion machinery and gD are necessary and sufficient to mediate cell-cell fusion [42], but other glycoproteins gM, gE, and gI, and the non-glycosylated membrane protein UL45 can regulate syncytium formation [43]. Cell-cell fusion induced by HSV is also negatively modulated by the glycoprotein K (gK) which regulates the core fusion machinery when co-expressed with gB, gH/gL, and gD [44]. Similarly, the glycoprotein gM downregulates surface expression of both gD and gH/gL heterodimer, inhibiting HSV-1-induced cell-cell fusion [45,46]. gK and UL20 protein form a functional protein complex that physically interacts with gB, thereby modulating the fusogenic properties gB [47]. Finally, the tegument proteins UL11, UL16, and UL21 can form a complex tightly associated with gE on its cytoplasmic tail, to modulate its function for cell-cell fusion [48]. Along with the core fusion machinery, the glycoproteins gE/gI heterodimer is also involved in HSV-induced cell-cell fusion and required for MGC formation [43,44,45,46,47,48,49].

Whereas the gD receptors (HVEM, and nectin-1 or nectin-2) are required for HSV mediated cell-cell fusion [50,51], heparan sulfate appears less important for cell-cell fusion than for viral entry [51,52]. It remains elusive how interaction of gD with its entry receptors leads to cell-cell fusion. One hypothesis is that the binding of gD with entry receptors induces conformational changes of gD, enabling its interaction with the core fusion machinery and activation of its fusogenic activity. Other cellular receptors for gB and/or gH/gL may also exist, and the binding of these glycoproteins to these additional receptors could also trigger fusion activity, and bypass the requirement for gD. The paired immunoglobulin-like type 2 receptor (PILR) has been identified as an entry coreceptor that associates with gB, and interactions between PILR and gB are involved in membrane cell-cell fusion events during HSV-1 infection [53]. Interestingly, the cellular protein tyrosine phosphatase 1B (PTP1B) has been recently reported to be specifically required for the cell-cell fusion by HSV-1 and not for cell-free virus infection [54].

Human cytomegalovirus (HCMV) infects a broad range of cell types, such as epithelial cells, endothelial cells, fibroblasts, leukocytes, monocytes, and macrophages [55]. As usual, the ability of HCMV to enter into these different cell types requires the coordinated interactions of viral glycoproteins with multiple host receptors [56]. It has been proposed that HCMV can use different forms of gH/gL heterodimer, including a trimeric complex with the gO glycoprotein and a pentameric complex with UL128 to UL131 proteins, to engage distinct receptors, and this engagement then triggers gB-mediated membrane fusion [57]. In vivo, HCMV spreading is largely cell-associated, and clinical isolates have been shown to induce enlarged flower-shaped syncytial foci in vitro [10,58]. In contrast, laboratory-adapted viral strains spread via diffusion of cell-free virus particles and fail to recapitulate the phenotypic characteristics of virus clinical isolates due to rapid acquisition of genetic mutations [59,60]. The ability of wild-type HCMV to spread via cell-to-cell route through cell-cell fusion can be attributed to the high level expression of the pentameric complex gH/gL/UL128-131, and this cell-to-cell spreading process is resistant to neutralizing antibodies and can overcome specific host restriction factors [60]. Similar to HSVs, HCMV gB and gH/gL are sufficient to mediate cell-cell fusion, and this process is most efficient at neutral pH [61]. Overexpression of gB promotes cell-cell fusion [62,63], whereas gB lacking the cytoplasmic domain fails [64]. In contrast, a single amino acid mutation within gB can promote cell-cell fusion and syncytium formation [65]. Recently, it has been reported that gB-mediated cell fusion and syncytia formation can be potently inhibited by neutralizing monoclonal antibodies (mAbs) specifically targeting the antigenic domain 5 (AD-5) in the ectodomain of gB, which has been shown to possess the predicted fusion loops [66]. In addition, the glycoprotein gO has been involved in HCMV-induced cell-cell fusion, and anti-gO antibodies can also block syncytium formation [67].

### 1.2. Paramyxoviridae

The *Paramyxoviridae* family contains several human viruses capable of inducing cell-cell fusion, including Sendai, Nipah, and Hendra viruses, as well as the Measles virus (MV) and the human respiratory syncytial virus (HRSV) [68,69,70,71]. Paramyxoviruses are spherical, enveloped, viruses with a linear negative-strand RNA genome encoding the nucleocapsid protein (N), the phosphoprotein (P), the matrix protein (M), the fusion protein (F), the large protein (L), and the binding transmembrane glycoprotein (G), which may have a hemagglutinin activity (also named H), associated or not with a neuraminidase activity (called HN). Here, we will focus on Measles virus and respiratory syncytial virus, which are the viruses of this family that have been the most studied for inducing cell-cell fusion and syncytia formation, in vitro, but especially in vivo, in infected patients.

Measles is a highly contagious disease that affects children and young adults, usually causing skin rashes, but severe complications can progress to fatal encephalitis. Measles virus (MV) is an enveloped virus of the Morbillivirus genus that first infects epithelial cells but also dendritic cells and alveolar macrophages of the respiratory tract before spreading by cis-infection to lymphocytes in lymph nodes and finally neurons [72,73,74,75]. Virus replication begins with the attachment of the viral envelope protein Hemagglutinin (H) to different receptors (CD46, Signaling Lymphocyte-Activation Molecule (SLAM), also named SLAMF1 or CD150, and Nectin-4) [76]. The wild-type strain of MV preferentially recognizes SLAM which is expressed on both B and T cells, while attenuated vaccine MV strains uses the CD46 receptor widely expressed on all cells [77]. Finally, nectin-4, a cellular adhesion protein of epithelial cell junctions, acts as a co-receptor for virus entry and can be used by both wild type and attenuated vaccine viruses [78]. The virus entry is mediated by both hemagglutinin and the fusion F protein. Hemagglutinin is organized on tetramers at the surface of virions, and is composed of a hydrophobic fusion peptide, two heptad repeat regions A and B (HRA/HRB, respectively) and a transmembrane domain [79]. After binding to the receptor, hemagglutinin induces a coordinated series of conformational changes of the different domains of the F protein, and the final F conformation triggers insertion of the fusion peptide in the host cell membrane, leading to the fusion between virions and the target cell [80], by a pH-independent process at the plasma membrane [79].

MV, but also other Morbilliviruses able to infect dogs, cats, cattle, seals, and cetaceans, are also characterized by their capacity to induce cell-cell fusion, both in vitro and in vivo. The formation of multinucleated cells is one of the major landmark in the development of measles, and has been observed in lymph nodes, respiratory tract and thymus after MV infection [81,82,83]. In 1973, White and Boy already observed syncytia of infected human thymocytes in lymphoid tissues of infected patients [83]. Similarly, the formation of multinucleated giant cells following MV infection of lymphocytes was initially observed in vivo, in African infected children [84]. Multinucleated giant cells were also initially detected within epidermal cells of Measles skin lesions by electron microscopy [85]. The in vivo MV-infected giant cells are able to produce infectious viral particles and can contribute to viral dissemination in patients [86]. In vitro, MV-induced syncytia have been mainly studied in epithelial cells, but formation of syncytia was also observed in vitro between infected human dendritic cells. Cell-cell fusion was only observed when DCs were activated by lymphocytes through CD40 engagement [87]. More recently, a new study demonstrated that the MV-induced cell-cell fusion leads to an increase of the IFN-I response in infected human epithelial cells and mature dendritic cells, via the nuclear translocation of IRF-3 [70]. In most cell-culture assays used to study MV-induced cell-cell fusion, this process is governed by interaction of viral F and H proteins with the cellular receptors CD46 and SLAM [88]. However, infected epithelial cells, which do not express the SLAM receptor, can form syncytia [89], indicating that the mechanisms by which cell-cell fusion is triggered in MV-infected cells still need better characterization. Interestingly, Kelly et al. [90] have shown in vitro that the formation of syncytia between infected HEK293T cells, a human embryonic kidney cell-line, expressing the cellular receptor SLAM, was drastically reduced when the lipid raft-associated Tetherin/BST2 protein was overexpressed. Tetherin/BST2 is a cellular restriction factor that restricts replication of numerous viruses, including HIV-1, by clustering viral particles at the cell surface and inhibiting virion release [91]. By using truncation mutants, they showed that the modification of the C-terminal glycosyl-phosphatidylinositol (GPI) anchor of BST2 was sufficient for significant decrease of cell-cell fusion and syncytium formation through targeting of the H protein [90].

Human respiratory syncytial virus (HRSV) is one of the most prevalent viral pathogen responsible for infant lower respiratory diseases, commonly bronchiolitis, but complications can lead to severe pneumonia [92]. HRSV primarily infects superficial airways and type I alveolar epithelial cells [93]. In vivo, infection of epithelial cells leads to the formation of large syncytia, hallmark of the cytopathic effect of HRSV [94]. Unlike other *Paramyxoviridae* as MV, HRSV genome codes for three envelope proteins: the Fusion protein (F), the attachment glycoprotein (G), and the small hydrophobic protein (SH), all involved in virus entry into host target cells [95]. The G protein is a highly glycosylated membrane protein that binds directly to heparin sulfate proteoglycans (Glycosaminoglycans GAGs), as well as to the CX3C-chemokine Receptor 1 (CX3CR1) on the apical surface of polarized epithelial cells [96]. The F protein also facilitates the attachment step by interacting directly with cellular heparan sulfate [97]. Following attachment, a fusion process is realized by the viral F fusion protein, allowing entry of the viral genome into host cells. However, two distinct mechanisms are still debated to explain virus entry. Krzyzaniak et al. [98] reported that in vitro infection of human epithelial HeLa cells was related to an actin-dependent internalization of viruses, which penetrate through Rab5-positive endocytosis vesicles, suggesting a macropinocytosis mechanism for virus entry. The viral F protein is then cleaved by the protease Furin, leading to its fusion with endosomal membranes for release of the viral genome into the cytoplasm [98]. The second proposed mechanism is a fusion process occurring directly at the plasma membrane [99]. In this process, the virus attaches itself on cholesterol microdomains of lipid rafts at the surface of human bronchial epithelial cells. This leads to local cytoskeletal rearrangements through the activation of the Pak1 kinase, resulting in the fusion between the virus and the membrane [99]. These reports indicate that the fusion protein F plays a central role in HRSV-mediated syncytium formation, and it was recently suggested that accumulation of the F protein on cell surface of infected cells helped the binding to the plasma membrane of neighboring uninfected cells. The rapprochement of the two membranes would be followed by the creation of a membrane fusion pore, thanks to the fusogenic activity of the F protein, which allows the formation of multinucleated fused cells for efficient spreading of HRSV [100]. In addition to the viral glycoproteins, cellular factors may be considered in the formation of syncytia mediated by HRSV. Interestingly, Gower et al. [101] demonstrated that the small GTPase RhoA was essential for HRSV-induced cell-cell fusion through modulation of the formation of microvilli at the cell surface [102,103].

### 1.3. Coronaviridae

*Coronaviridae* are a family of viruses first characterized in humans in late 1960s which has recently become an increasingly severe threat to global public health, exemplified by the previous outbreaks of Severe Acute Respiratory Syndrome CoV (SARS-CoV) in 2001 and Middle East Respiratory Syndrome CoV (MERS-CoV) in 2012, but especially by the current COVID-19 pandemic induced by SARS-CoV-2 which is responsible for hundreds of thousands of people deaths worldwide [104]. CoVs are enveloped viruses with a positive single-strand RNA genome of around 30 kb in length that encodes for four main viral proteins, the spike protein (S) involved in viral entry, the membrane protein (M), the envelope protein (E) required for virus assembly and release, and the nucleocapsid proteins (N) [105]. The CoV family consists of four sub-families (α-, β-, γ-, and δ-CoVs), among which α-CoVs and β-CoVs mainly tend to infect mammals, while γ-CoVs and δ-CoVs prefer to infect birds and fishes, but some can also infect mammals [106]. α-CoVs include two of the seven human CoVs known to infect humans, such as HCoV-229E and HCoV-NL63, mainly responsible of upper respiratory diseases with symptoms of the common cold but also the porcine epidemic diarrhea virus (PEDV), while β-CoVs include SARS-CoV, MERS-CoV, and SARS-CoV-2, sharing between 80% and 50% genetic similarity and responsible for the development of severe respiratory diseases, as well as two other human CoV (HCoV-OC43 and HCoV-HKU1) also mainly responsible of common cold that can also advance to severe pneumonia and bronchiolitis, in addition to the mouse hepatitis virus (MHV) [107,108]. Finally, γ- and δ-CoVs are mainly involved in fish and bird infections, and they include the avian infectious bronchitis virus (IBV) which can be responsible for severe global economic losses in the poultry industry, but some can also infect mammals, such as the porcine *delta*-CoV (PDCoV) that causes serious vomiting and diarrhea in suckling piglets [106,109,110,111,112].

The S spike viral protein plays the critical role for virus entry into target cells, and is the primary determinant of the host tropism and pathogenesis of CoVs. It is a type I highly glycosylated transmembrane protein, which inspired the name “coronavirus” due to its crown-like (corona) shape. The ectodomain of all CoV S proteins share the same organization in two domains: an N-terminal domain named S1 that is responsible for receptor binding at the surface of the target cells, and a C-terminal S2 domain responsible for fusion between the viral envelope and the host-cell membranes that can take place at the plasma membrane or in acidic endosomal compartments after internalization [113,114]. Although the S protein conformational changes can be mainly primed by receptor binding, they can also need additional triggers, such as proteolytic activation or pH acidification, in endosomal compartments [115]. The virus-cell fusion process begins with cleavage of the S protein by cellular proteases that yields the receptor binding domain of S1 and the fusion domain of S2, which is sometimes subsequently again cleaved to generate and expose the fusion peptide directly involved in fusion of the viral and cellular membranes [116,117,118]. CoVs can recognize different cell surface molecules as their host cell receptors, including proteins, sugars, and heparan sulfate depending of viral sub-families and the receptor binding domains (RBD) of the S spike [104]. Remarkably, CoVs usually can adapt and change receptors for virus cellular entry in the new host either by genetic mutation or recombination to infect a new host species [119]. In addition, CoVs are even capable of recombination when two viruses are infecting the same cell at the same time [119].

The ability of many CoVs, including animal viruses, to mediate cell-cell fusion to form MGCs (or syncytia) has long been well documented, both in vitro and in vivo, but we will focus here on the three human MERS-CoV, SARS-CoV, and especially SARS-CoV-2 which are responsible for the development of severe and acute respiratory diseases and currently constitute the main threat to public health linked to CoV infections. The presence of infected large syncytia has been definitively observed in vivo in lung tissues of infected patients, as evidenced in the large majority of post-mortem lung samples of individuals who died of severe respiratory diseases induces by these three human CoVs. As expected, this cell-cell fusion process and MGC formation can be recapitulated in vitro in cell-culture systems [120,121] and is a direct consequence of the high fusogenic activity of the viral Spike protein [122,123,124,125].

As mentioned, recent reports show that infected syncytia are frequently observed in lung tissues of SARS-CoV-2-infected patients with acute respiratory syndrome, and immunohistochemistry analyses have identified that these MGCs mainly originate from cell-cell fusion of alveolar epithelial cells. In addition, these SARS-CoV-2-mediated syncytia could also derive from cell-cell fusion of infected alveolar macrophages, target cells that certainly play a crucial role for the systemic hyper-inflammation known as “cytokine storm” (or macrophage activation syndrome) observed in patients with critical disease manifestations [123,124,125,126,127,128,129,130,131,132]. Some authors even suggest that abnormal cellular syncytia are hallmarks of COVID-19 lung pathology, and it is obvious that some of these giant cells correspond to syncytial histiocytic cells as confirmed by positive CD68 expression, a specific marker of myeloid cells, including monocytes and macrophages [127]. However, the efficiency of cell-cell fusion mainly depends, at least in vitro, on viral isolates and host cell-types. For example, SARS-CoV-2 causes extensive MGCs formation in Vero cells, a simian kidney epithelial cell-line largely used to isolate CoVs in vitro, whereas relatively few cell-cell fusion events are triggered by SARS-CoV in the same cell-type [133].

Entry into target cells of MERS-CoV relies on binding of the S protein with the surface receptor dipeptidyl peptidase 4 (DPP4) expressed on lung epithelial cells and is then primed by cellular proteases (TMPRSS2 and members of the cathepsin family) which function as activators of the S protein [134,135]. Similarly, in vitro studies show that interaction between the S protein expressed on virus-donor cells and the DPP4 cell surface receptor, as well as priming and activation of the S protein by the cellular TMPRSS2 enzyme, are directly involved in the cell-cell fusion process and MGC formation mediated by MERS-CoV. This cell-cell fusion process can be blocked in vitro by antibodies targeting DPP4 and by the camostat mesylate, a specific inhibitor of the cellular TMPRSS2 protease [133,134,135,136,137,138]. In addition, the S protein of MERS-CoV seems more efficient than the SARS-CoV S protein for triggering cell-cell fusion of infected cells [136]. Interestingly, cell-cell fusion of MERS-CoV-infected epithelial cells is totally blocked by treatment with the camostat inhibitor, whereas virus entry and infection by cell-free virus particles are only partially inhibited [138], suggesting that the enzymatic activity of TMPRSS2 plays a critical role in MERS-CoV-mediated cell-cell fusion but has a lower impact on cell-free virus infection.

SARS-CoV and SARS-CoV-2 both utilize ACE2 (Angiotensin converting enzyme 2) as host receptor to enter into lung type II epithelial target cells (also called pneumocytes) [115,116,117,125,126,132,139,140,141]. Like for MERS-CoV, priming and activation of the S protein involve serine proteases, such as TMPRSS2, for exposure of the S2 domain fusion peptide required for fusion of viral and cellular membranes, as well as for triggering cell-cell fusion between neighboring target cells [121,142,143,144,145,146,147,148]. In addition, the S protein of SARS-CoV-2 and MERS-CoV contains, unlike the SARS-CoV S protein, an additional multibasic cleavage site used by the cellular serine protease furin before subsequent activation by TMPRSS2, thus promoting cell-cell fusion and virus cell-to-cell spreading in lung cells [148]. Interestingly, it has been reported that the receptor binding domain of the SARS-CoV-2 S1 subunit binds to ACE2 with a significant higher affinity than the SARS-CoV receptor binding domain [146,147], which may facilitate cell-free virus entry of SARS-CoV-2 into target cells, but also a higher ability to trigger cell-cell fusion of infected cells compared to SARS-CoV. The higher fusogenic activity of the SARS-CoV-2 S2 subunit, compared to that of SARS-CoV, also contributes to this higher ability to mediate cell-cell fusion [149]. Similarly, the S protein of MERS-CoV seems also more efficient than the SARS-CoV S protein for triggering cell-cell fusion of infected cells [136]. Some recent in vitro studies, performed on transfected cell-lines, confirm that infected virus-donor cells expressing the SARS-CoV-2 S spike protein can fuse with adjacent non-infected cells expressing ACE2 and TMPRSS2, leading to the formation of giant syncytia [120,121,148,149,150]. This syncytium formation process is thus totally inhibited by blocking antibodies targeting the ACE2 receptor, as well as by the camostat mesylate, the specific inhibitor of the TMPRSS2 protease [120,121]. Interestingly, a recent report indicates that some cellular factors such interferon-induced transmembrane proteins (IFITMs 1, 2, and 3), a family of restriction factors that block entry of many viruses, including HIV-1, negatively modulate syncytium formation through inhibition of the cell-cell fusion process mediated by the S spike protein [120]. However, other currently submitted studies report controversial results showing rather a positive influence of endogenous IFITMs proteins to promote cell-free SARS-CoV-2 infection, as well as syncytia formation in a human lung epithelial cell-line [151]. It is therefore critical to reproduce these observations and perform further investigations using more relevant target cells of SARS-CoV-2, including lung epithelial cells or primary myeloid cells, such as macrophages, to decipher, at the cellular and molecular levels, the mechanisms, as well as the viral and cellular factors, that govern, and may control and modulate, syncytium formation mediated by these three human CoVs.

### 1.4. Retroviridae

*Retroviridae* are a family of enveloped viruses whose genome is formed of two linear copies of single-stranded positive RNA of about 7–11 kb that needs to be reverse-transcribed into a proviral DNA that will be subsequently integrated into the host cell genome [152]. All retroviruses contain at least three major genes encoding the main virion structural components synthesized as polyprotein precursors, Gag, Pol and Env. After proteolytic maturation, these three precursors will give rise, respectively, (i) to the capsid, matrix and nucleocapisd proteins; (ii) the viral reverse-transcriptase, protease and integrase enzymes involved in essential steps of the viral life cycle; and (iii) the viral envelope glycoproteins involved in virus entry in their host target cells. In addition to virus structural proteins and enzymes, some retroviruses also code for small proteins with regulatory functions.

Retroviral infections can cause a wide spectrum of diseases in their respective hosts ranging from malignancies to immune deficiencies and neurologic disorders. A few human retroviruses have been identified, but only the human immunodeficiency viruses (HIVs) and the human T-lymphotropic viruses (HTLVs), responsible for development of severe immunodeficiency, malignant or neurological diseases, are thus of clinical importance and will be discussed here. As evidenced in vitro, these two human retroviruses can induce cell-cell fusion between infected virus-donor cells and uninfected neighboring target cells, leading to the formation of MGCs or syncytia. However, the presence of infected MGCs have been observed in vivo only in tissues of HIV-1-infected patients, whereas such observations are still missing regarding HTLV-1 infection.

To date, four types of HTLVs, as well as four types of related simian T-lymphotropic viruses have been identified, but HTLV-1 has been the most studied and is responsible for the development of adult T-cell leukemia/lymphoma and a demyelinating disease called HTLV-associated myelopathy also known as tropical spastic paraparesis [153,154,155,156]. HTLV-1 is present in clusters of high endemicity, such as in Japan, equatorial Africa, the Caribbean, and South America [157,158]. Although HTLV-1 primarily infects CD4+ T lymphocytes, other cell types have been found to contain proviral HTLV-1 DNA in vivo, including CD8+ T lymphocytes, B lymphocytes, as well as myeloid cells, such as monocytes, macrophages, and dendritic cells. As other retroviruses, the envelope glycoproteins of HTLV-1 involved in virus entry in the target cells is derived from an envelope precursor that is cleaved by cellular proteases yielding the surface subunit (gp46) non-covalently-associated with the transmembrane subunit (gp21). The first step of viral infection relies on successive binding of the surface gp46 to heparan sulfate proteoglycans (HSPGs) [159], neuropilin-1 (NRP-1) [160], and the glucose transporter type 1 (GLUT-1) [161], expressed on target cells. Then, the transmembrane gp21 mediates fusion of the viral envelope with the membrane of the target cell [157,158]. However, HTLV-1 particles are usually not detected in blood of infected patients, and cell-free virions produced in vitro are very poor or even not infectious, indicating that HTLV-1 does not spread between its target cells by a cell-free mechanism but primarily uses cell-to-cell processes for virus dissemination. While the most recent studies regarding HTLV-1 cell-to-cell transfer focused on the formation of the so-called “virological synapse” for intercellular virus dissemination [158], it was suggested by previous reports that cell-cell fusion between infected virus-donor cells and target cells leading to syncytium formation could be the main mechanism for HTLV-1 intercellular dissemination [162]. In vitro, it is evident that infected cells expressing the viral envelope glycoproteins at the cell surface can fuse with neighboring uninfected cells expressing the different receptors involved in virus entry, leading to syncytium formation that will subsequently die by apoptosis [162,163,164,165,166,167,168,169]. Through their role in establishment and maintenance of tight intercellular junctions, adhesions molecules, such ICAM proteins, expressed at the cell of infected cells also help to trigger cell-cell fusion and syncytium formation by interacting with integrins expressed on target cells [162,165,166,167,168]. By contrast, the tetraspanin CD82, a cellular membrane protein that facilitates cellular adhesion, could rather inhibit HTLV-1-mediated syncytium formation through interaction with the viral envelope [170]. Interestingly, it has been also reported that HTLV-1 virions accumulate at the plasma membrane of infected cells in a biofilm-like extracellular viral platform that resembles a bacterial biofilm [171]. This viral biofilm can be rapidly transmitted from infected to neighboring uninfected target cells but could also facilitate cell-cell fusion and syncytium formation. However, to our knowledge, all these results have been described in vitro, and there is to date no evidence for the presence of infected syncytia in HTLV-1-infected patients. Therefore, these in vitro observations absolutely need to be corroborated with in vivo studies that could be performed in relevant animal models, such as STLV-infected monkeys, in order to demonstrate the presence of HTLV-1-induced syncytia during the natural course of infection, as it was observed in HIV-1-infected patients and simian immunodeficiency virus(SIV)-infected macaques.

**Human immunodeficiency viruses**, the etiological agents of AIDS, are classified into two main types: HIV-1 and HIV-2 [152]. While HIV-1 is largely prevalent worldwide, HIV-2 is more closely related the simian immunodeficiency viruses, but is less pathogenic in humans, and is mostly confined to West Africa. Here, we are referring only to the literature regarding HIV-1 because the huge majority of the data available regarding HIV-mediated formation of infected MGCs are related to HIV-1, even if the presence of infected MGCs have been definitively demonstrated in vivo in tissues of SIV-infected monkeys.

HIV-1 targets cells of the immune system expressing the CD4 receptor and one of the chemokine co-receptor, CXCR4 and/or CCR5, for virus entry [172]. Through expression of these receptors and coreceptors, CD4+ T lymphocytes and cells of the myeloid lineage, including macrophages, dendritic cells, as well as bone osteoclasts, are the main target cells of HIV-1 recognized by the viral envelope, initially produced as a precursor (gp160), and then proteolytically cleaved into two subunits: the surface (gp120) and transmembrane (gp41) subunits that remain non-covalently associated and oligomerize as trimers on the surface of virus particles. The surface gp120 initially binds to CD4 and then to the chemokine receptor on the surface of susceptible cells, leading to subsequent conformational changes of the transmembrane gp41 that finally mediates fusion of the viral membrane with the target cell membrane. The viral envelope also determines the cellular tropism of the viral strains that can be classified in 3 types: CXCR4-using T cell-tropic, CCR5-using T cell-tropic (also called non-macrophage-tropic), and macrophage-tropic strains using either CCR5, CXCR4, or both [173]. In addition to this cell-free infection process, it is now well established, at least for infection of CD4+ T cells, that HIV-1 can be transmitted and spread through direct cell-to-cell transfer, and this route of infection may be the predominant mode of propagation in infected patients [174]. HIV-1 can take advantage of the specific particularities and functions of its immune cell targets, and subvert intercellular communications to allow infection through a multiplicity of intercellular structures and membrane protrusions, like tunneling nanotubes, filopodia, or lamellipodia-like structures. Other features of immune cells, like the immunological synapse or the phagocytosis properties, can be also hijacked by HIV-1 and used to infect CD4+ T cells or myeloid target cells. Finally, HIV-1 can use the fusogenic capacity of its envelope glycoproteins to provoke fusion between infected donor cells and neighboring target cells to form infected syncytia (or MGCs) with high capacity of viral production and improved capacities of motility or survival. All these modes of cell-to-cell transfer are now considered as viral mechanisms to escape immune system and antiretroviral therapies and could be involved in the establishment of persistent virus reservoirs in different host tissues [174].

Cell-cell fusion between HIV-1-infected CD4+ T cells and uninfected CD4+ T cells has been initially proposed to be another mechanism for HIV-1 infection and dissemination between T cells [175,176,177,178,179]. This cell-cell fusion is mainly mediated through interaction between envelope glycoproteins expressed at the cell surface of infected cells and CD4 and co-receptors expressed on target cells [175,176,177,179,180], even if adhesin/integrin molecules expressed on virus-donor cells, as well on target cells, facilitate and maintain intercellular contacts for efficient cell-cell fusion. Several groups thus proposed that the cytopathic effect related to the formation of T-cell syncytia could be a mechanism for the CD4+ T cells loss observed in HIV-1-infected patients [175,176,181]. However, formation of T-cell syncytia has been a controversial subject since other studies did not observe formation of T-cell syncytia using HIV-1-infected primary CD4+ T cells [182,183,184], and it has been suggested that these giant syncytia could be in vitro artifacts only observed with immortalized cell-lines and restricted to CXCR4-viruses [185]. However, small T-cell syncytia, containing no more than five nuclei, have been then observed in vivo in lymph nodes from HIV-1-infected patients [186]. In addition, some more recent studies using HIV-1-infected “humanized mouse” experimental models reported the presence of motile infected syncytia in lymph nodes of infected animals but smaller than those observed in vitro [187]. These motile small T-cell syncytia can establish tethering interactions with uninfected T cells that may facilitate virus cell-to-cell transmission and spreading to neighboring target cells in lymphoid tissues [187,188].

While the process of T-cell syncytia formation has been largely documented in vitro and discussed for its in vivo relevance, some recent reports indicate that these processes of cell-cell fusion for HIV-1 infection and dissemination are not restricted to T cells since infected multinucleated macrophages, as well as multinucleated DCs, can be found in vivo in tissues of HIV-1-infected patients [189,190]. Myeloid cells, such as macrophages and DCs, are indeed poorly infected by cell-free viruses because of the high expression of host cell restriction factors, such as sterile alpha motif and HD-domain–containing protein 1 (SAMHD1), an enzyme that cleaves deoxynucleoside triphosphates (dNTPs) and depletes the pool of intracellular nucleotides necessary for efficient HIV-1 replication in these non-cycling myeloid cells. Therefore, virus cell-to-cell transfer trough cell-cell fusion of myeloid cells may likely represent a dominant mode of virus dissemination in vivo and may allow for productive infection of these cell types [191,192]. Macrophages have been proposed to participate in virus dissemination and establishment of persistent virus reservoirs in numerous host tissues, including lymph nodes, spleen, lungs, genital and digestive tracts, and the central nervous system (CNS) [193,194,195,196,197]. Virus access to the CNS is indeed mainly related to the migration of infected perivascular monocytes/macrophages through the blood brain barrier and can result in a massive infiltration of infected macrophages often detected as infected MGCs [196,198,199,200,201,202,203,204,205]. Similarly, the presence of HIV-1-infected syncytia derived from DCs and expressing specific markers of DCs and T cells was found at the surface of the nasopharyngeal tonsils, adenoids and parotid glands of HIV-1-infected patients [206,207,208]. While several groups showed the presence of infected multinucleated macrophages or DCs in tissues, and, more specifically, in the brain of HIV-1-infected patients and monkeys experimentally infected with SIVs, the cellular and molecular mechanisms related to this MGC formation remained poorly investigated [175,176,177,178,179,180,181,182,183,184,185,186,187,188,189,190,191,192,193,194,195,196,197,198,199,200,201,202,203,204,205,206,207,208,209]. Interestingly, we have recently revealed that HIV-1 uses a specific and common two-step cell-cell fusion mechanism for virus transfer and dissemination from infected CD4+ T lymphocytes to myeloid target cells, including macrophages, immature DCs, and osteoclasts [191,192,210]. In the first step, infected T cells establish contacts with macrophages, OCs or DCs, resulting in the fusion with these myeloid cell targets for virus transfer. Then, the newly formed Gag+ fused cells acquire the ability to fuse with neighboring uninfected macrophages, DCs or OCs, leading to the formation of infected MGCs that can survive for a long time, at least in vitro, and produce high level of fully infectious virus particles. These two sequential cell-cell fusion processes are dependent on interactions of the viral envelope expressed on virus-donor T cells with the CD4 and chemokine co-receptors expressed on myeloid target cells, since they are inhibited by anti-CD4 antibodies, as well as by drugs, such as T20, a specific fusion inhibitor targeting the transmembrane gp41 subunit, and also by co-receptor antagonists. In addition, the viral auxiliary Nef protein is also involved in promoting cell-cell fusion of HIV-1-infected macrophages, and thus participates in the formation of HIV-1-induced MGCs. Importantly, despite their large size, these infected MGCs migrate faster than their infected mononucleated counterparts, and may facilitate virus dissemination in tissues [211]. These recent studies indicate that this two-step cell-cell fusion process leading to the formation of highly virus-productive MGCs allows to bypass the restriction imposed by some host cell restriction factors, such as SAMHD1 [192], and may represent, compared to cell-free virus infection, an original and largely more efficient mode of virus transmission for viral spreading in myeloid cells and may be a major determinant for virus dissemination in vivo. These infected MGCs could indeed survive for a long time in host tissues to produce infectious virus particles as shown in vivo in lymphoid organs and especially in the CNS of HIV-1-infected patients and SIV-infected monkeys [198,199,200,201,202,203,204,208,209,210,211,212,213]. Similarly, the first step related to the initial cell-cell fusion of infected CD4 T cells with myeloid cell targets agrees with results showing that myeloid cells from lymphoid tissues of SIV-infected macaques contain T-cell markers and viral RNA and DNA originating from infected T cells [189,214,215,216,217,218]. However, more investigation is needed to determine the roles of HIV-1-induced MGCs in pathogenesis and whether these myeloid cell-derived MGCs may participate in vivo in HIV-1 cell-to-cell transmission, dissemination, and formation of persistent virus reservoirs in lymphoid and non-lymphoid tissues of infected patients.

### 1.5. Other Viruses

**Hepatitis C virus (HCV)** is an enveloped virus belonging to the *Hepacivirus* genus of the *Flaviridae* family [219], and its genome is a 10 kb monopartite, linear, positive single-stranded RNA. HCV primarily infects human hepatocytes, causing chronic hepatitis, hepatic cirrhosis, and hepatocellular carcinoma [220]. During replication, the viral RNA is translated into a polyprotein and then processed into three structural proteins, including the Envelope glycoproteins E1 and E2 which form a E1/E2 heterodimer, and seven nonstructural proteins [221]. The structural viral proteins form the capsid and envelope, while the non-structural proteins assume the enzymatic activities required for efficient replication [222]. After a facultative initial recognition of the cellular LDL receptor (LDL-R) at the surface of hepatocytes, HCV enters its target cells through clathrin-mediated endocytosis [223]. This process requires interaction of the envelope E2 glycoprotein with the cellular scavenger receptor B-I (SR-BI), triggering conformational changes for subsequent binding to the tetraspanin CD81 as co-receptor [224]. The tight junction proteins Claudin-1 and Occludin are also required for this process, but do not interact directly with the HCV envelope glycoproteins. E1 and E2 are N-glycosylated transmembrane proteins assembled as a heterodimer, stabilized by disulfide bonds on infected cells [225,226]. E2 first attaches to the SR-BI receptor, driving rearrangements of lipoproteins found associated with viral particles that expose the receptor binding sites of CD81 [227]. Only E2 has the ability to bind the receptors SR-BI and CD81 [228], while E1 is needed to trigger conformational modifications of the E1-E2 complex, allowing access of the E2 binding sites to SR-BI and CD81 [229]. Following the attachment step, virus particles and CD81-claudin-1 complexes are internalized through endocytosis [224]. The fusion between the viral particle and the early endosomal membranes is then governed by both E1 and E2 [230,231]. While endocytosis-mediated entry appears to be a predominant mode of infection for cell-free virions, some in vitro studies have shown that HCV could also infect target cells by cell-to-cell contacts, and this mode of transmission can sometimes involve cell-cell fusion, leading to the formation of syncytia [232,233,234]. The fusogenic activity of the HCV envelope glycoproteins for triggering cell-cell fusion was previously described [235]. The first study that described HCV-mediated cell-cell fusion and syncytia formation was performed on the human B lymphoblastoid TO-FE cell-line [236]. These authors showed that HCV-infected cells first form intercellular bridges, followed by cell-cell fusion leading to the formation of syncytia, and finally to cell lysis of this syncytia and virus release. Then, another group also observed the HCV-mediated formation of syncytia in vitro, using hepatocyte-derived carcinoma Huh7.5 cells transfected with HCV RNA [237], and this was related to the disruption of autophagy by HCV. Despite these few in vitro observations, evidences for HCV-mediated cell-cell fusion is still discussed [238], and there is actually very poor evidence in vivo for such HCV-triggered syncytium formation, questioning the relevance of these observations for HCV pathogenesis. To our knowledge, the presence of multinucleated giant cells has been finally reported in HCV-infected patients in only a single publication, in which Miccheli et al. [239] found multinucleated giant hepatocytes in 2.6% of 856 liver biopsies of HCV-infected patients. Of note, some of these patients were diagnosed as co-infected with HCV and HIV-1, but some others were only HCV-infected.

**Ebola viruses (EboVs)** belong to the *Filoviridae* family of enveloped viruses with a negative single-stranded RNA genome. Five species of EboVs have been identified and are mainly responsible for severe and often fatal hemorrhagic fevers in humans and non-human primates [240]. The Zaire EboV species is responsible for the outbreaks of lethal hemorrhagic fevers that took place in Africa during the last decade [241]. EboV can infect several cells types, including endothelial cells, hepatocytes, fibroblasts, dendritic cells, macrophages, and monocytes [240]. The virus binds to the host target cells using the viral glycoprotein GP, a heterodimer that consists in a receptor binding subunit GP1 and a fusion subunit GP2 [242]. No specific receptor for EboV has been identified to date, and GP1 can recognize and bind to several surface proteins on target cells, depending of the cell-lines, such as the C-type lectins DC-SIGN (Dendritic Cell-Specific Intercellular adhesion molecule-3-Grabbing Non-integrin) and hMGL (human N-acetylgalactosamine-specific C-type lectin), on macrophages and endothelial cells [243,244]; the tyrosine kinase receptor Axl on a large scale of primary mammalian cells except on B lymphocytes [245]; some β-integrins expressed at the surface of the human kidney 293T cell-line [246]; and the T lymphocyte immunoglobulin and mucin domain 1 (TIM-1), mainly expressed on mucosal epithelial cells and also known as the cell surface receptor of the hepatitis A virus [247]. Following attachment through recognition of these cell surface molecules, the cell-free viral particle is internalized by micropinocytosis in early endosomal vesicles [248]. In late acidic endosomes, the GP1/GP2 heterodimers is cleaved through activation by cysteine proteases, cathepsins B and L, leading to conformational changes [249,250,251]. The receptor binding domain of GP1 is thus exposed to bind to the late endosomal protein Neinmann-Pick 1 (NPC-1), a cholesterol transporter protein playing a critical role for virus entry, and thus triggers fusion with endosomal membranes trough the fusion activity of the GP2 subunit [252]. The EboV core is finally released in the cytoplasm of the host cell to start viral replication.

In addition to this intracellular virus-endosome membrane fusion process, it was reported that EboV was also able to induce cell-cell fusion [253]. In this study, the authors co-cultured human HeLa epithelial cells transfected with the viral GP glycoprotein as virus-donor cells, and HeLa cells expressing the β-galactosidase reporter gene to detect the cytoplasmic exchanges following cell-cell fusion with target cells. In this co-culture system, the cell-cell fusion required optimal low pH expositions of the GP-expressing donor cells before co-culture [253]. Moreover, neutralizing antibodies targeting GP inhibited syncytia formation, showing the essential role of the GP protein for cell-cell fusion [253,254]. Similarly, Markosyan and colleagues [255] used COS7 cells, an African green monkey fibroblast cell-line, expressing the viral GP protein as a virus donor cell, and co-cultured with human HEK293T target cells, and confirmed that cell-cell fusion was mediated by the EboV GP through the same mechanism of EboV-induced endosomal fusion. After cleavage in endosomal compartments, the cell-cell fusion process requires recycling of the GP glycoproteins back to the cell surface. Since the cell-cell fusion induced by EboV has been poorly described, the mechanisms underlying this fusion process remain unclear and its role on viral transfer and spreading of Ebola virus in vivo remain uncertain, since no reports were published, to our knowledge, regarding the presence of EboV-induced syncytia in infected patients.

**Orthoreoviruses and Aquareoviruses** belong to the *Reoviridae* family and are non-enveloped viruses with a segmented double-strand RNA genome [256]. Interestingly, some members of these two genera are the only known examples of non-enveloped viruses able to induce syncytium formation in vitro, but also in vivo, to promote virus cell-to-cell spreading as a major determinant of virulence in infected hosts [257,258]. Syncytium formation is directly involved in direct virus cell-to-cell transmission, but it also participates in rapid release of intracellular progeny virions through subsequent disruption of syncytia and thus contributes to the pathogenicity of these viruses in their natural hosts. While Aquareoviruses displaying fusogenic properties to trigger syncytium formation have been isolated from fishes, mollusks, and crustaceans, Orthoreoviruses with fusogenic properties have been isolated from birds and reptiles, but also from mammals, including baboons and bats [256,257,258,259]. Moreover, several fusogenic Orthoreoviruses from bat origin have been identified in association with severe acute respiratory infections or meningo-encephalitis in humans [260,261,262,263,264,265,266], indicating that these viruses may emerge as zoonotic infections potentially responsible for future outbreaks. The intriguing property of these non-enveloped viruses to mediate syncytium formation for virus cell-to-cell transfer and dissemination between host target cells is related to expression of an original family of viral fusogens, the fusion-associated small transmembrane (FAST) proteins, initially discovered by Roy Duncan and colleagues [257,258,259,260,261,262,263,264,265,266,267,268]. Functionally, Reovirus FAST proteins largely differ from the 3 well-characterized classes of enveloped virus fusion proteins to trigger cell-cell fusion [1]. Fusogenic Aquareoviruse and Orthoreoviruses genomes indeed contain an additional gene encoding for a FAST protein which is not found in the genomes of the non-fusogenic Reoviruses [257,258]. As non-structural, FAST proteins are not directly involved in virus entry and are non-essential for virus replication. Expressed at the cell surface of infected host cells and localizing at adherent cell-cell junctions, such as epithelial cells and fibroblasts, fusogenic Reovirus FAST proteins are sufficient to induce syncytium formation. FAST proteins are able to induce cell-cell fusion in a wide range of cell-types. Structurally, FAST are small integral transmembrane proteins, ranging from 100–200 amino acids in size, with a short N-terminal ectodomain which could function as a fusion peptide to insert on the lipid bilayers of cell membranes, a single transmembrane domain, and a rather large C-terminal cytoplasmic tail of about 40 to 140 residues [257]. The three ecto-, transmembrane-, and cytoplasmic-domains of the FAST proteins are actively required for efficient cell-cell fusion, but how each domain is involved in the cell-cell fusion process to promote syncytium formation is still unclear, even if cellular cofactors have been involved in the FAST-induced cell-cell fusion process. While calcium-dependent cadherin proteins have been involved in the initial stage of cell-cell attachment [269], Annexin 1 (AX1) is required for post-fusion events, such as pore expansion [270]. In addition, both early and late stages of the cell-cell fusion process also involve active remodeling of the actin cytoskeletton [269].

## 2. Concluding Remarks

As reviewed here, it is evident that some viruses can use, at least in in vitro experimental systems, the fusogenic capacity of some viral proteins expressed at the surface of infected cells to trigger cell-cell fusion between infected virus-donor cells and neighboring target cells to form enlarged multinucleated cells and syncytia. The presence of such virus-induced syncytia has definitively been evidenced in host tissues and organs during the natural course of infections for several families of enveloped viruses, including major human virus pathogens, such as HIV-1, SARS-CoV-2, and Herpesviruses, as well as some members of the *Paramyxoviridae* (i.e., Measles virus and the respiratory syncytial virus). However, for most of these viruses, in vivo data regarding the role and functions of these syncytia in virus spreading, virulence, and virus persistence during the natural course of infection in their natural hosts are still discussed.

While all enveloped viruses express proteins displaying fusogenic features and should be theoretically able to induce cell-cell fusion, only certain enveloped viruses can trigger cell-cell fusion in tissues of infected hosts, indicating that host cellular pathways and mechanisms that maintain cell mobility, cellular adhesion, and cell membrane integrity certainly play specific roles in cell-cell fusion and syncytium formation, since fusion involves direct cell-to-cell contacts, cytoskeleton rearrangements, and the mixing of cellular contents, including membranes, cytoplasm, organelles, and nuclei. It is therefore critical to develop relevant in vitro experimental systems using primary host target cells for a better understanding of the cellular and molecular mechanisms, as well as the viral and cellular factors, that govern, and may control and modulate, virus-induced syncytium formation. Finally, further development of relevant experimental animal models should also help to in vivo investigation of the roles and functions of virus-induced syncytia in virus transmission and dissemination, as well as in virus evasion to innate and adaptive immunity, for a better understanding of their contribution to pathogenesis.
